# Unintended pregnancy: Risk factors and perinatal outcomes in a nulliparous population

**DOI:** 10.1002/pmf2.70043

**Published:** 2025-06-24

**Authors:** Rebecca J. Post, Luke Schmidt, Brian A. Crosland, Oluwatoni Okuyemi, Jenny Chang, Argyrios Ziogas, Robert M. Silver, Samuel Parry, George R. Saade, William A. Grobman, Brian M. Mercer, Hyagriv N. Simhan, Caitlin Bernard, Judith H. Chung

**Affiliations:** ^1^ Department of Obstetrics and Gynecology University of California Irvine Medical Center Orange California USA; ^2^ Department of Obstetrics and Gynecology Oregon Health and Science University Portland Oregon USA; ^3^ Department of Medicine University of California Irvine Medical Center Orange California USA; ^4^ Department of Obstetrics and Gynecology University of Utah Salt Lake City Utah USA; ^5^ Department of Obstetrics and Gynecology University of Pennsylvania Philadelphia Pennsylvania USA; ^6^ Department of Obstetrics and Gynecology University of Texas Medical Branch Galveston Texas USA; ^7^ Department of Obstetrics and Gynecology Northwestern University Chicago Illinois USA; ^8^ Department of Obstetrics and Gynecology Case Western Reserve University The MetroHealth System Cleveland Ohio USA; ^9^ Department of Obstetrics, Gynecology, and Reproductive Sciences University of Pittsburgh School of Medicine Pittsburgh Pennsylvania USA; ^10^ Department of Clinical Obstetrics and Gynecology Indiana University Indianapolis Indiana USA

**Keywords:** adverse pregnancy outcomes, health disparities, mental illness, nulliparous, pregnancy intention, unintended pregnancy, unplanned pregnancy

## Abstract

**Background:**

Previous studies have found that unintended pregnancies are associated with late prenatal care and adverse pregnancy outcomes. Unintended pregnancy in nulliparous individuals who establish early prenatal care is of particular interest because outcomes in these pregnancies are not confounded by outcomes from prior births or late initiation of prenatal care.

**Objective:**

The purpose of this study is to investigate maternal risk factors and perinatal outcomes associated with unintended pregnancies in a nulliparous population who established early prenatal care.

**Study design:**

This is a secondary analysis of the Nulliparous Pregnancy Outcomes Study: Monitoring Mothers‐to‐be (nuMoM2b), a prospective cohort study in which 10,038 nulliparous individuals were followed throughout their pregnancy from the first trimester. Participants who indicated their pregnancy intention on a standardized questionnaire were included in the analysis. Maternal characteristics and pregnancy outcomes of those reporting a planned pregnancy were compared with those reporting an unplanned pregnancy. Multivariable logistic regression with stepwise model selection was used to identify maternal characteristics associated with unplanned pregnancy.

**Results:**

A total of 10,020 participants in the nuMoM2b study identified their pregnancies as either “planned” (58.5%, *n* = 5,859) or “unplanned” (41.5%, *n* = 4161). Compared with those having a planned pregnancy, those with unplanned pregnancies were more likely to be younger, non‐Hispanic Black or Asian, not married, of lower socioeconomic status, and to report alcohol or tobacco use prior to pregnancy, even after adjusting for covariates (all *p* values ≤ 0.04). Those with unplanned pregnancies were not more likely to have a history of mental illness. After controlling for potentially confounding factors, only treatment of mental illness during pregnancy or postpartum remained significantly associated with unplanned pregnancy (adjusted odds ratio 1.29, 95% confidence interval 1.01–1.64, *p* = 0.04).

**Conclusions:**

Our findings suggest that nulliparas who received early prenatal care for an unplanned pregnancy did not have more frequent adverse perinatal outcomes compared to planned pregnancies; however, they received treatment more frequently for mental illness during pregnancy and postpartum.

## 1 INTRODUCTION

Approximately half of the pregnancies in the United States are unplanned [1, 2, 3]. Unintended pregnancies have been associated with adverse health outcomes including late entry to prenatal care, preterm birth, fetal growth restriction, low birthweight infants, and increased risk of maternal mental health problems [4, 5, 6, 7]. Additionally, there are significant disparities in rates of unintended pregnancy by race, ethnicity, education, and socioeconomic status [8, 9]. There are also notable public health costs as a result of unintended pregnancy, estimated to be $11 billion in maternity and infant care, not accounting for the costs of abortion care and additional care required due to adverse perinatal outcomes [10]. However, it is unclear whether population differences in those that have unplanned pregnancies, including risk of late entry to prenatal care, could account for the increased risk of adverse pregnancy outcomes rather than pregnancy intention itself. The purpose of this study is to investigate maternal risk factors and perinatal outcomes associated with unintended pregnancies in a nulliparous population who established early prenatal care.

## 2 MATERIALS AND METHODS

This was a secondary analysis of the Nulliparous Pregnancy Outcomes Study: Monitoring Mothers‐to‐be (nuMom2b), a prospective cohort study that followed 10,038 nulliparous gravidas (with no prior pregnancy 20 weeks’ gestation or greater) throughout their pregnancies, which occurred between 2010 and 2013. Detailed study methodology is published elsewhere [11]. In brief, subjects were recruited from hospitals affiliated with eight clinical centers across the United States. Each site's local governing institutional review board(s) approved the nuMoM2b protocol and procedures. Participants had four study visits during pregnancy, the first occurring in the first trimester from 6 0/7 to 13 6/7 weeks’ gestation until time of delivery, with information collected from personal interviews, self‐administered questionnaires, clinical measurements, medical record abstraction, and biological specimens. Each subject was surveyed regarding the intentionality of the current pregnancy (planned or unintended). For the purposes of the current analysis, only those participants who reported their pregnancy intention were included.

Maternal characteristics and delivery outcomes of gravidas with unintended pregnancies were compared to subjects with planned pregnancies in a bivariate analysis. Maternal characteristics and demographics examined included maternal age, self‐reported race and ethnicity, body mass index, gravidity, marital status, insurance type, level of education, family income, poverty level, alcohol consumption or tobacco use in 3 months prior to pregnancy, illegal drug use, chronic hypertension, diabetes, asthma, and mental illness treated prior to pregnancy. Mental illness included depression, anxiety, posttraumatic stress disorder, bipolar disorder, schizophrenia/schizoaffective disorder, or other mood disorders. The subjects’ survey response to the question, “Is this pregnancy desired?” with “yes” or “no” was also included, as the “wantedness” of a pregnancy is distinct from pregnancy intention. Pregnancy outcomes evaluated included type of labor (spontaneous, augmented, induced, or cesarean delivery without labor), cesarean delivery, postoperative complications such as deep venous thrombosis, pulmonary embolus, or readmission to hospital within 14 days, severe anemia treated with blood transfusion, hypertensive disorders of pregnancy including gestational hypertension, preeclampsia, superimposed preeclampsia, eclampsia, HELLP (Hemolysis, Elevated Liver Enzymes, and Low Platelets) syndrome, placental abruption, mental illness treated during pregnancy or 14 days postpartum, and maternal death. Fetal and neonatal outcomes included pregnancy outcome (live birth, stillbirth, fetal demise, elective, or indicated termination), abnormal amniotic fluid such as oligohydramnios or polyhydramnios, fetal growth restriction defined as birthweight below the 10th percentile, preterm delivery before 37 weeks, 1‐min and 5‐min APGAR score less than 7, neonatal intubation following delivery, neonatal intensive care unit (NICU) admission, and neonatal death. Given rarity of neonatal complications, a neonatal complication composite score was also utilized in this study (1‐min or 5‐min APGAR less than 7, neonatal intubation, NICU admission, and/or neonatal death).

All statistical analyses were performed on SAS 9.4 (SAS Institute). Statistical significance was set at *p* < 0.05, using two‐tailed tests. In bivariate analysis, chi‐square tests or Fisher's exact tests were used to assess the association of risk factors and adverse pregnancy outcomes with unintended pregnancy. To examine maternal characteristics associated with unintended pregnancy, adjusted odds ratios (aORs) and 95% confidence intervals (CIs) were estimated using multivariable logistic regression. Final model was selected through stepwise model selection, with entry and stay significance levels set at ≤0.15. To assess the association between unintended pregnancy and adverse pregnancy outcomes, additional multivariable logistic regression models were fitted for each outcome, adjusting for maternal social‐demographic and clinical covariates. For each adverse outcome, the aOR and 95% CI for unintended pregnancy were reported.

## 3 RESULTS

Of the 10,038 nulliparous individuals who participated in the nuMoM2b study, 10,020 of the participants identified their pregnancies as either “unintended” or “planned” and were included in the analysis; 18 indicated that they were “unsure” about their pregnancy intention and were therefore excluded from the analysis. Among those meeting inclusion criteria, 58.5% (*n* = 5859) of gravidas reported an intended pregnancy, and 41.5% (*n* = 4161) of subjects reported an unintended pregnancy. Table 1 summarizes the maternal characteristics and demographic characteristics of the two study groups. As compared with their counterparts, those with an unintended pregnancy were more likely to be younger, self‐reported to be Black or Hispanic, overweight or obese, unmarried, and respond “no” to the survey question asking if the pregnancy was desired. They were also more likely to smoke or consume alcohol in 3 months prior to pregnancy, report illicit drug use, have government insurance, preexisting medical conditions, lower‐income level, and less educational attainment (all *p* values < 0.003). No differences were seen in rates of mental illness prior to pregnancy between the two groups (*p* = 0.12). Table 2 summarizes the aORs of maternal characteristics associated with unplanned pregnancy. After adjusting for covariates, the following maternal characteristics associated with unintended pregnancies remained statistically significant: younger maternal age, non‐Hispanic Black or Asian/Other race/ethnicity, not married, gravidity ≥ 2, education less than completed college, family income < $50,000, and alcohol or tobacco use reported in 3 months prior to pregnancy (all *p* values ≤ 0.04).

**TABLE 1:** Demographics and maternal characteristics by pregnancy intention.

Maternal characteristics	Unintended pregnancy, *n* = 4161 (41.5%)	Intended pregnancy, *n* = 5859 (58.5%)	*p*‐value^a^
Maternal age (years)	24.0 (±5.4)	29.1 (±4.8)	<0.0001
Maternal age category			<0.0001
≤19	9255 (22.2%)	159 (2.7%)	
20–29	2554 (61.4%)	2885 (49.2%)	
30–39	638 (15.3%)	2715 (46.3%)	
40+	44 (1.1%)	100 (1.7%)	
Race/ethnicity^b^			<0.0001
Non‐Hispanic White	1742 (41.9%)	4245 (72.5%)	
Non‐Hispanic Black	1076 (25.9%)	342 (5.8%)	
Hispanic	965 (23.2%)	730 (12.5%)	
Asian/Other	378 (9.1%)	542 (9.3%)	
Ethnicity			<0.0001
Not Hispanic	3195 (76.8%)	5129 (87.5%)	
Hispanic	965 (23.2%)	730 (12.5%)	
BMI category			<0.0001
Underweight or normal weight	1893 (45.5%)	3330 (56.3%)	
Overweight	1038 (24.9%)	1406 (24.0%)	
Obese	1111 (26.7%)	1061 (18.1%)	
Is the pregnancy desired?			<0.0001
Yes	4018 (96.6%)	5817 (99.3%)	
No	143 (3.4%)	42 (0.7%)	
Gravidity			0.40
1	3069 (73.8%)	4365 (74.5%)	
≥2	1092 (26.2%)	1492 (25.5%)	
Marital status			<0.0001
Not married	3151 (75.7%)	926 (15.8%)	
Married	1010 (24.3%)	4933 (84.2%)	
Insurance			<0.0001
Commercial insurance	1544 (37.1%)	3692 (63.0%)	
Government insurance	1991 (47.8%)	717 (12.2%)	
Others	626 (15.0%)	1450 (24.7%)	
Education			<0.0001
≤High school	1494 (35.9%)	490 (8.4%)	
Some college	1662 (39.9%)	1288 (22.0%)	
Completed college	684 (16.4%)	2088 (35.6%)	
>College	316 (7.6%)	1991 (34.0%)	
Family income			<0.0001
<$50,000	1648 (39.6%)	1146 (19.6%)	
$50,000–$99,999	671 (16.1%)	1712 (29.2%)	
$100,000+	446 (10.7%)	2504 (42.7%)	
Poverty			<0.0001
>200% of fed poverty level	1230 (29.6%)	4430 (75.6%)	
100%–200% of fed poverty level	630 (15.1%)	539 (9.2%)	
<100% of fed poverty level	904 (21.7%)	392 (6.7%)	
Chronic hypertension	130 (3.1%)	113 (1.9%)	0.0001
Diabetes			<0.0001
Pregestational diabetes	88 (2.1%)	63 (1.1%)	
Gestational diabetes	154 (3.7%)	241 (4.1%)	
Asthma	832 (20.0%)	968 (16.5%)	<0.0001
Mental illness^c^	705 (16.9%)	924 (15.8%)	0.12
Alcohol consumption^d^	2535 (60.9%)	3939 (67.2%)	<0.0001
Tobacco use^d^	1183 (28.4%)	598 (10.2%)	<0.0001
Illegal drug use	1498 (36.0%)	1935 (32.9%)	0.0021

*Note*: Data are *n* (±standard deviation or %).

Abbreviation: BMI, body mass index.

^a^ *p*‐value from chi‐square test or Fisher's exact tests for categorical variables and two sample *t* test for continuous variables.

^b^ Race and ethnicity were self‐reported.

^c^ Mental illness treated prior to pregnancy.

^d^ Alcohol or tobacco use in 3 months prior to pregnancy.

**TABLE 2:** Adjusted odds ratios of maternal characteristics associated with unintended pregnancy.

Maternal characteristic	Adjusted odds ratio and 95% CI^a^			*p*‐value
Maternal age category				
≤19	Referent			
20–29	0.50	0.40	0.61	<0.0001
30–39	0.31	0.24	0.40	<0.0001
40+	0.43	0.27	0.68	0.0003
Race/ethnicity				
Non‐Hispanic White	Referent			
Non‐Hispanic Black	1.66	1.40	1.97	<0.0001
Hispanic	1.14	0.99	1.32	0.072
Asian/Other	1.36	1.13	1.64	0.0011
Marital status				
Not married	Referent			
Married	0.15	0.13	0.17	<0.0001
Gravidity				
'1	Referent			
'2+	0.78	0.69	0.88	<0.0001
Education				
≤High school	Referent			
Some college	1.14	0.97	1.34	0.11
Completed college	0.82	0.68	0.99	0.040
>College	0.56	0.45	0.70	<0.0001
Family income				
<$50,000	Referent			
$50,000–$99,999	0.67	0.58	0.78	<0.0001
$100,000+	0.47	0.39	0.56	<0.0001
Alcohol consumption^b^				
No	Referent			
Yes	1.30	1.14	1.49	0.0001
Unknown	0.77	0.64	0.92	0.0041
Tobacco use^b^				
No	Referent			
Yes	1.44	1.24	1.66	<0.0001

Abbreviation: CI, confidence interval.

^a^ All characteristics in Table 1 were included in the full model. Stepwise model selection used significant level of entry = 0.15 and significant level of stay = 0.15. Adjusted ORs for each covariate listed in this table were from final model after selection.

^b^ Alcohol or tobacco use in 3 months prior to pregnancy.

Table 3 compares maternal and perinatal outcomes according to pregnancy intention. Study participants with an unintended pregnancy were statistically more likely to have induced labor, postoperative complications, severe anemia, hypertensive disorders of pregnancy (gestational hypertension, preeclampsia, superimposed preeclampsia, eclampsia, or HELLP), mental illness treatment during pregnancy or postpartum, fetal growth restriction, delivery before 37 weeks, 5‐min APGAR score less than 7, need for neonatal intubation, neonatal death, and/or stillbirth (all *p* values < 0.05).

**TABLE 3:** Pregnancy outcomes by pregnancy intention.

Pregnancy outcomes	Unintended pregnancy, *n* = 4161 (41.5%)	Intended pregnancy, *n* = 5859 (58.5%)	*p*‐value^a^
Maternal outcomes			
Type of labor			<0.0001
Spontaneous labor	635 (15.3%)	1043 (17.8%)	
Augmented labor	1606 (38.6%)	2315 (39.5%)	
Induced labor	1442 (34.7%)	1806 (30.8%)	
Cesarean without labor	200 (4.8%)	412 (7.0%)	
Cesarean delivery	1078 (25.9%)	1545 (26.4%)	0.91
Postoperative complications	87 (2.1%)	79 (1.3%)	0.0041
Severe anemia	79 (1.9%)	66 (1.1%)	0.0014
Hypertensive disorders			
Gestational hypertension	993 (23.9%)	1287 (22.0%)	0.019
Preeclampsia	365 (8.8%)	382 (6.5%)	<0.0001
Superimposed preeclampsia	304 (7.3%)	333 (5.7%)	0.0008
Eclampsia	304 (7.3%)	333 (5.7%)	0.0009
HELLP	1 (0.0%)	10 (0.2%)	0.032
Placental abruption	28 (0.7%)	55 (0.9%)	0.15
Mental illness treatment^b^	345 (8.3%)	407 (6.9%)	0.012
Maternal death	1 (0.0%)	0 (0.0%)	0.42
Fetal/neonatal outcomes			
Pregnancy outcomes			0.049
Live birth	3895 (93.6%)	5597 (95.5%)	
Stillbirth	33 (0.8%)	21 (0.4%)	
Fetal demise < 20 weeks	38 (0.9%)	49 (0.8%)	
Elective termination	5 (0.1%)	10 (0.2%)	
Indicated termination	10 (0.2%)	15 (0.3%)	
Abnormal amniotic fluid	245 (5.9%)	300 (5.1%)	0.095
Fetal growth restriction	133 (3.2%)	142 (2.4%)	0.02
Preterm delivery < 37 weeks	444 (10.7%)	493 (8.4%)	<0.0001
One‐minute APGAR < 7	455 (10.9%)	592 (10.1%)	0.078
Five‐minute APGAR < 7	98 (2.4%)	106 (1.8%)	0.037
Need for neonatal intubation	93 (2.2%)	95 (1.6%)	0.017
NICU admission	21 (0.5%)	34 (0.6%)	0.67
Neonatal death	20 (0.5%)	12 (0.2%)	0.014

Abbreviations: APGAR, Appearance, Pulse, Grimace, Activity, and Respiration Score; HELLP, Hemolysis, Elevated Liver Enzymes, and Low Platelet Count; NICU, neonatal intensive care unit.

^a^ *p*‐value from chi‐square test or Fisher's exact test for categorical variables and two sample *t* test for continuous variables.

^b^ Mental illness treated during pregnancy through 14 days postpartum.

**FIGURE 1:**
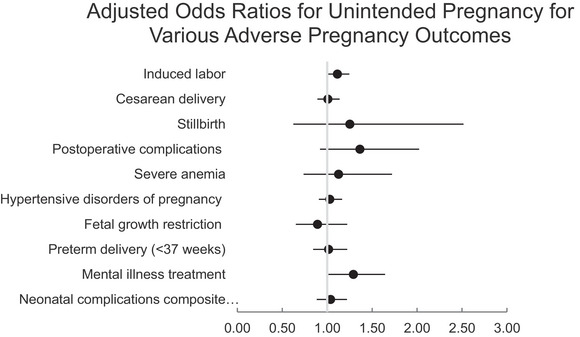
Forest plot summarizing adjusted odds ratios of adverse pregnancy outcomes among nulliparas with unintended pregnancy.

Figure 1 is a forest plot summary of the aORs from the multivariable analyses for the adverse pregnancy outcomes associated with unintended pregnancies (see Table S1 for numeric values). After adjusting for potential confounders (multivariable logistic models control for all covariates in Table 1), study participants who reported their pregnancy as unintended had higher odds of having treatment for mental illness during pregnancy or in the first 2 weeks of the postpartum period (aOR 1.29, 95% CI 1.01–1.64, *p* = 0.04). However, there were no significant statistical differences regarding the remainder of pregnancy outcomes between the two groups, including induced labor, cesarean delivery, stillbirth, postoperative complications, severe anemia, hypertensive disorders of pregnancy, fetal growth restriction, delivery before 37 weeks, or neonatal complications composite score (1‐min or 5‐min APGAR less than 7, neonatal intubation, NICU admission, neonatal death).

## 4 DISCUSSION

Unintended pregnancy was prevalent in our cohort (41.5%), comparable to the estimated rate of 45% in the United States based on data from the National Survey of Family Growth in 2011 [2]. Those with an unplanned pregnancy in our study were more likely to be younger, not married, non‐Hispanic Black, of lower socioeconomic status, and to report alcohol, tobacco, or illegal drug use, even after adjusting for covariates—these findings are consistent with prior studies examining the maternal risk factors for unintended pregnancy [2, 8, 9, 12]. In our bivariate analysis, when compared to planned pregnancies, unintended pregnancy was found to be associated with several adverse pregnancy outcomes, such as fetal growth restriction, preterm delivery, stillbirth, hypertensive disorders of pregnancy, etc. However, after controlling potential confounders given the population differences, we did not find unintended pregnancy to be associated with adverse pregnancy outcomes except notably for increased risk of receiving mental health treatment during pregnancy or postpartum.

Previous studies have suggested that unintended pregnancies are at higher risk for adverse pregnancy complications, such as low birthweight and preterm birth [4–8, 13, 14]. However, these studies may not have adequately accounted for confounding factors. Those who become pregnant unintentionally are more likely to initiate prenatal care later in the pregnancy, have lower socioeconomic status, and preexisting medical conditions than those who planned their pregnancy [2, 8, 9, 12]. Therefore, it can be unclear if these perinatal complications stem from pregnancy intention itself, or are due these other associated maternal factors, such as the delay in prenatal care, socioeconomic variables, or presence of health conditions. For instance, Shah et al. published a systematic review of 15 retrospective studies and surveys, which demonstrated increased odds of low birthweight and preterm delivery among those with unintended pregnancies in an unadjusted analysis of the combined studies’ raw data. However, there were notable heterogeneities among these studies and 8 of the 15 studies did not adjust for any confounding factors. The confounding factors that seven of the studies controlled for varied greatly with only four of them that specifically controlled for prenatal care [13]. Furthermore, it is known that establishing early and routine prenatal care can affect pregnancy outcomes, and is a separate issue from pregnancy intention [15, 16]. Our study has not only taken into account these known population differences, but has also utilized a patient population that has never carried a pregnancy beyond 20 weeks (nulliparous) and has established early and presumably regular prenatal care (given the requirement of enrollment in the first trimester and regular follow‐up required in the nuMoM2b study), thus eliminating bias from prior pregnancies or lack of prenatal care.

Unintended pregnancy has also been linked to mental health issues [17, 18, 19, 20]. Qiu et al. conducted a meta‐analysis of 30 case‐control and cohort studies and found unintended pregnancy to be significantly associated with the development of postpartum depression [19]. We similarly found in our study that those with unplanned pregnancies were more likely to receive treatment for mental health during pregnancy or postpartum compared to those with a planned pregnancy, though there were no differences in presence of baseline mental health disorders between these two groups prior to pregnancy. This finding underscores the importance of providing mental health support for these pregnant individuals, in addition to improving access to preconception counseling and reliable contraception, given that nearly half of all pregnancies in the United States are unplanned [1–3, 12]. Additional research is needed to gain a better understanding of why individuals with unintended pregnancies seek more mental health care, identify opportunities to improve diagnosis and psychiatric care of those with unintended pregnancies, and to determine methods to prevent mental health issues in people who become unexpectedly pregnant. It is also unclear whether the unplanned pregnancy itself leads to increased need for psychiatric treatment, or individuals with more severe pre‐existing mental health issues are more prone to experiencing unplanned pregnancies.

This secondary analysis has several strengths, including the utilization of a large database of individuals from multiple different sites across the United States who were followed prospectively from early pregnancy until delivery and the postpartum period. Pregnancy intention was collected at the time of study enrollment in the first trimester, thus reducing the risk of recall bias that may occur in retrospective surveys. Some studies have shown that gravidas may underreport unintended pregnancy when asked about intention retrospectively [21, 22]. An additional strength of this study was that all participants in the nuMoM2b study database were having their first singleton birth. This excluded heterogeneity in reproductive life stage and number of previous children, which otherwise could affect the relationships between unintended pregnancy and perinatal outcomes. Furthermore, this study analyzed a population of pregnant individuals who established early prenatal care by virtue of enrolling in the nuMoM2b study, and thus eliminated late initiation of prenatal care as a potential confounding factor for adverse pregnancies outcomes.

This study also has its limitations. The survey utilized in the nuMoM2b study dichotomizes pregnancy into “intended” or “unintended” based on a single question. There are several reasons why a pregnancy may be unplanned. For this reason, some studies have disaggregated the “unintended” category into pregnancies that are mistimed or unwanted, resulting in differential effects and determinants [4]. The nuMoM2b study did include a survey question of whether the pregnancy was desired. However, an overwhelming majority of the study participants reported the pregnancy was desired (96.6% in the unintended group and 99.3% of the intended group) and the elective termination rates were exceedingly low (0.1%–0.2%) compared to rates typically observed in the general population [23]. This is likely due to selection bias as those with undesired pregnancies are less likely to enroll in a research study following women throughout their pregnancy and suggests that most of the study participants reported the pregnancy was unintended due to mistiming. Furthermore, the nuMoM2b study did not go into detail of what types of mental health treatments were more likely sought in those with unintended pregnancies, or what mental health diagnoses were being treated and their severity prior to pregnancy. Additional studies are needed to better understand the mental health‐care needs of those who become unexpectedly pregnant.

Our findings from this secondary analysis of a large prospective study suggest that nulliparas who receive early prenatal care for an unintended pregnancy do not have more frequent adverse perinatal outcomes after adjusting for potentially confounding variables; however, they more frequently receive treatment for mental illness during pregnancy and postpartum. These findings highlight the importance of ensuring accessible, timely prenatal care and mental health support for those with unexpected pregnancies. Furthermore, the conclusions drawn from this analysis suggest that health disparities of at‐risk populations, such as those studied in this manuscript, need further study. The “intendedness” of a pregnancy, alone, does not appear to be an independent risk factor for poor perinatal outcomes.

## CONFLICT OF INTEREST STATEMENT

The authors declare no conflicts of interest.

## Supporting information



Supporting Information

## Data Availability

Data from the parent nuMoM2b study are currently publicly available through the Eunice Kennedy Shriver National Institute of Child Health and Human Development (NICHD) National Institutes of Health Data and Specimen Hub (DASH; https://dash.nichd.nih.gov/).
