# Supplementary material for: Unintended pregnancy: Risk factors and perinatal outcomes in a nulliparous population
Source: Pregnancy (Hoboken). 2025 Jun 24;1(4):e70043. doi: 10.1002/pmf2.70043 (PMC13344660; doi:10.1002/pmf2.70043)
Supplement: Supplementary file 1 [file PMF2-1-e70043-s001.docx]

**Supplementary Table S1:** Adjusted odds ratios of pregnancy outcomes among nulliparas with unintended pregnancy

| **Pregnancy outcomes** | **Adjusted odds ratio (95% CI)^a^** | **p-value** |
| --- | --- | --- |
| Induced labor | 1.11 (0.99-1.25) | 0.07 |
| Cesarean delivery | 1.00 (0.89-1.14) | 0.92 |
| Postoperative complications | 1.36 (0.92-2.02) | 0.12 |
| Severe anemia | 1.12 (0.74-1.72) | 0.58 |
| Hypertensive disorders of pregnancy^b^ | 1.02 (0.91-1.17) | 0.66 |
| Mental illness during pregnancy or postpartum^c^ | 1.29 (1.01-1.64) | 0.04 |
| Stillbirth | 1.25 (0.62-2.52) | 0.53 |
| Fetal growth restriction | 0.89 (0.65-1.22) | 0.48 |
| Delivery less than 37 weeks | 1.02 (0.84-1.22) | 0.87 |
| Neonatal complications composite score^d^ | 1.04 (0.88-1.22) | 0.65 |

*^a^ Multivariable logistic models control for all covariates in Table 1*

*^b^ Includes gestational hypertension, pre-eclampsia, superimposed pre-eclampsia, eclampsia*

*^c^ Mental health illness treated during pregnancy through 14 days postpartum*

*^d^ Composite include one-minute APGAR < 7, five-minute APGAR < 7, neonatal intubation, NICU admission, neonatal death*
